# Brain insulin resistance: a key pathological hub linking metabolic and neuropsychiatric comorbidities

**DOI:** 10.3389/fnagi.2026.1716291

**Published:** 2026-03-09

**Authors:** Shanwen Tang, Yifan Liao, Maoyi Yang, Rensong Yue

**Affiliations:** 1 Hospital of Chengdu University of Traditional Chinese Medicine, Chengdu, China; 2 Chengdu University of Traditional Chinese Medicine, Chengdu, China

**Keywords:** Alzheimer’s disease, anxiety disorders, brain insulin resistance (BIR), depressive disorders, diabetes-associated cognitive decline

## Abstract

The high rate of comorbidity between metabolic diseases and neuropsychiatric disorders suggests a shared underlying pathogenic mechanism. However, the biological basis of this relationship remains unclear. This study aims to clarify the role of brain insulin resistance (BIR) in linking metabolic dysfunction to neuropsychiatric symptoms based on existing evidence. The analysis shows that BIR disrupts limbic system function through two primary molecular pathways: (1) impairment of the PI3K/Akt/mTOR pathway, which decreases the expression of synaptic plasticity-related proteins and causes deficits in long-term potentiation (LTP); (2) activation of the TLR4/MyD88 inflammatory axis, promoting pro-inflammatory cytokine release from glial cells. These changes result in characteristic neuropsychiatric phenotypes, including amygdala hyperactivity (emotional disorders), hippocampal atrophy (memory impairment), and decreased prefrontal cortex (PFC) function (executive dysfunction). This review highlights that interventions targeting BIR might simultaneously improve metabolic outcomes and neuropsychiatric symptoms, providing a theoretical foundation for trans-diagnostic treatment models. The findings support the view of BIR as a modifiable interface for metabolic- neuropsychiatric comorbidities and advocate for the development of a multidisciplinary collaborative framework to facilitate mechanism-based precision therapy.

## 1 Introduction

The global rise in type 2 diabetes (T2D) and obesity represents a major public health challenge (GBD 2021 Diabetes Collaborators, 2023). Their close associations with depressive disorders and mild cognitive impairment (MCI) further exacerbate health risks in metabolically compromised individuals (Chow et al., 2022). Consequently, understanding these relationships has become central to interdisciplinary research. Epidemiological data indicate that over 536 million people worldwide had T2D in 2021 (Sun et al., 2022). Among diabetic patients, the prevalence of depression is approximately 39.1% (Deng et al., 2024), while cognitive impairment affects as many as 45.0% (You et al., 2021), considerably higher than in the general population. A large-scale meta-analysis also demonstrated significantly increased risks of depression and suicidal tendencies (OR ≈ 1.4) among obese individuals, largely driven by the self-perception of being overweight (Haynes et al., 2019). This comorbidity pattern, “metabolic disorders associated with emotional disorders and cognitive decline,” not only markedly reduces patients’ quality of life but also decreases treatment adherence, raises medical costs, and results in poorer prognoses. Therefore, clarifying the underlying mechanisms is critical.

Is there a unified pathological mechanism underlying these comorbidities? Traditional research typically investigates individual disorders, such as depressive-like behaviors, anxiety traits, or memory deficits. However, this approach does not adequately explain why individuals with metabolic disorders frequently experience both emotional and cognitive symptoms. Recently, emerging clinical epidemiological, neuroimaging, and molecular biological evidence has identified BIR as a potential key intersection connecting metabolic disorders with various neuropsychiatric symptoms. Although this association has been initially confirmed, a comprehensive explanation of how BIR functions as a “pathological hub” that concurrently affects multiple disease pathways and phenotypes remains lacking. Further clarification and validation of the relevant evidence are also necessary.

This review introduces an integrated perspective of “BIR-induced psychiatric symptoms resulting from disruptions of multiple critical neural pathways.” The review aims to systematically address three scientific questions: (1) integrating multi-level evidence that BIR impairs neural function across metabolic and psychiatric disorders by damaging synaptic plasticity, activating neuroinflammation, disrupting nutrient sensing, and causing a hypothalamic-pituitary-adrenal (HPA) axis imbalance, thereby clarifying the central pathological role of BIR; (2) elucidating the molecular and structural basis of depression, anxiety, and cognitive decline co-occurrence, thus explaining the mechanism of metabolic-emotional/cognitive comorbidities; and (3) evaluating the therapeutic potential of interventions targeting BIR, including central-penetrant insulin sensitizers and lifestyle modifications, to simultaneously improve peripheral metabolic markers and neuropsychiatric symptoms, thereby providing theoretical and empirical support for a trans-diagnostic, multi-target therapeutic model.

## 2 Brain insulin and its pathological resistance

Recent studies indicate that insulin not only regulates peripheral glucose metabolism but also performs crucial physiological roles within the central nervous system (CNS). Insulin crosses the blood-brain barrier (BBB) and is extensively distributed in the brain (Rhea et al., 2018), particularly in regions associated with cognitive and emotional regulation, such as the hippocampus, hypothalamus, and PFC (Kellar and Craft, 2020). Insulin signaling modulates memory processes in the hippocampus (McNay et al., 2010), influences eating behaviors and peripheral metabolism via the hypothalamus (Brief and Davis, 1984), and affects synaptic plasticity, neuronal survival, neurotransmitter regulation, and energy homeostasis sensing. Specifically, insulin promotes synaptic plasticity and LTP by activating the PI3K/Akt pathway, thereby enhancing learning and memory functions (Zhao et al., 2019). Insulin also regulates metabolic activities in neurons and glial cells, maintains neuronal survival, and promotes neural stem cell proliferation and differentiation (Grillo et al., 2015). Additionally, insulin influences the synthesis and release of neurotransmitters such as dopamine and serotonin, regulating emotional and behavioral responses (Gruber et al., 2023).

Brain insulin resistance (BIR) is defined as a failure of the CNS to mount a normal response to insulin, resulting in abnormal activation or suppression of insulin signaling pathways (Alagiakrishnan and Halverson, 2024). Although specific causes of BIR remain incompletely understood, adverse consequences are prevalent across neuropsychiatric disorders. BIR is closely associated with cognitive impairment, emotional disorders, and neurodegenerative conditions, serving as a central pathological hub linking metabolic diseases and neuropsychiatric comorbidities.

## 3 Understanding molecular basis of BIR

### 3.1 Impairment of synaptic plasticity

Recent studies have demonstrated the impact of insulin signaling on molecular cascades underlying hippocampal plasticitiy, learning, and memory. BIR causes neuronal dysfunction and impaired synaptic plasticity primarily by inhibiting the PI3K/Akt/mTOR signaling pathway and overactivating GSK-3β kinase, ultimately leading to cognitive impairment and neurodegenerative diseases (AlRuwaili et al., 2025). The PI3K/Akt/mTOR pathway has long been recognized for its critical role in neuronal survival, synaptic plasticity, and neurotransmitter release. Insulin activates insulin receptors, subsequently activating the PI3K/Akt/mTOR pathway, promoting neuronal growth, synapse formation, and maintenance of function (Spinelli et al., 2020). Studies have demonstrated that insulin resistance (IR) significantly reduces Akt phosphorylation, inhibits mTORC1 activity, and decreases synaptic protein synthesis, further impairing synaptic plasticity (Spinelli et al., 2020). Additionally, inhibition of mTORC1 affects autophagy, causing accumulation of dysfunctional proteins and organelles, thereby exacerbating neuronal dysfunction (de Mello et al., 2019).

Overactivation of GSK-3β is another key mechanism contributing to impaired synaptic plasticity in BIR. Under healthy conditions, GSK-3β is inactivated by Akt, maintaining neuronal homeostasis. However, in BIR, Akt activity is blocked (FangFang Li et al., 2017), resulting in excessive dephosphorylation and activation of GSK-3β (Xu et al., 2018; Yamini et al., 2022). Overactivated GSK-3β promotes abnormal Tau protein phosphorylation, causing microtubule instability and neuronal apoptosis (Akhtar et al., 2020a,b). This is a characteristic pathological feature of Alzheimer’s disease (AD), where amyloid-beta (Aβ) deposition and hyperphosphorylated Tau form extracellular plaques and neurofibrillary tangles (Mei et al., 2023; Qiu et al., 2023). Additionally, GSK-3β affects the Wnt/β-catenin pathway by destabilizing β-catenin, impairing neuronal development and synaptic plasticity (Tian et al., 2021). Overactivation of GSK-3β is also closely related to neuroinflammation and mitochondrial dysfunction, further exacerbating neuronal damage (Akhtar et al., 2020b). A study on AD conducted in 2025 examined the PI3K/Akt/mTOR pathway in postmortem inferior parietal lobe tissue and found overactivation of the pathway, with elevated insulin receptor substrate 1 (IRS-1) and GSK-3β levels, indicating a connection between Aβ and the PI3K/Akt/mTOR axis (Tramutola et al., 2015). Moreover, IKKβ, JNK, ERK, mTOR, and S6K increase the serine phosphorylation of IRS under insulin-resistant conditions (Tanti and Jager, 2009), amplifying BIR and GSK-3β hyperphosphorylation, thereby accelerating AD progression (Wang et al., 2015).

Brain insulin resistance specifically damages the glutamatergic system via impaired ILK-GSK-3β signaling, reducing postsynaptic glutamate receptor trafficking and function, which causes synaptic deficits and memory loss (Shonesy et al., 2012). Normally, insulin exerts neuroprotective effects by reducing glutamate-induced intracellular Ca^2+^ elevation and superoxide production in rat cortical neurons and mitochondria (Pinelis et al., 2022). Notably, glutamate excitotoxicity is common in stroke and traumatic brain injury (Hou et al., 2025). Furthermore, BIR increases GluA1 palmitoylation through FoxO3a, impairing hippocampal synaptic plasticity and memory (Spinelli et al., 2017). Conversely, downregulating hippocampal insulin receptors or administering intranasal insulin reduces hippocampal glutamate synaptic concentration and increases glutamate efflux (Erichsen et al., 2025), alleviating cognitive impairment. Hippocampal-specific BIR induces basal dendritic atrophy, often accompanied by despair-like and anxiety-like behaviors, which are key factors in depression and anxiety (Reagan et al., 2021; Alves et al., 2025).

Brain insulin resistance also significantly affects monoamine neurotransmitter systems. BIR causes abnormal neurotransmitter release, particularly reduced glutamate and dopamine release, impairing neural transmission (Kellar and Craft, 2020; de Bartolomeis et al., 2023) and resulting in learning, memory deficits, and cognitive impairment (Grillo et al., 2015). Insulin-resistant animals exhibit impaired dopamine transporter activity, while dopamine D2 receptor mRNA expression increases under insulin deficiency conditions (de Bartolomeis et al., 2023). Neuron-specific insulin receptor knockout (NIRKO) mice exhibit BIR, elevated monoamine oxidase A and B (MAO-A/B), increased dopamine turnover, and subsequent anxiety- and depression-like behaviors (Kleinridders et al., 2015). Another study induced BIR in rats via intraventricular streptozotocin injection, resulting in AD-like manifestations including memory deficits, Aβ deposition, tau phosphorylation, and reduced dopamine and acetylcholine levels (Abosharaf et al., 2024). Similarly, Parkinson’s disease (PD) pathology involves abnormal α-synuclein (α-Syn) deposition in dopaminergic neurons of the substantia nigra pars compacta, causing neuronal apoptosis and dysregulation of PI3K/Akt/GSK-3β signaling (AlRuwaili et al., 2025).

**FIGURE 1:**
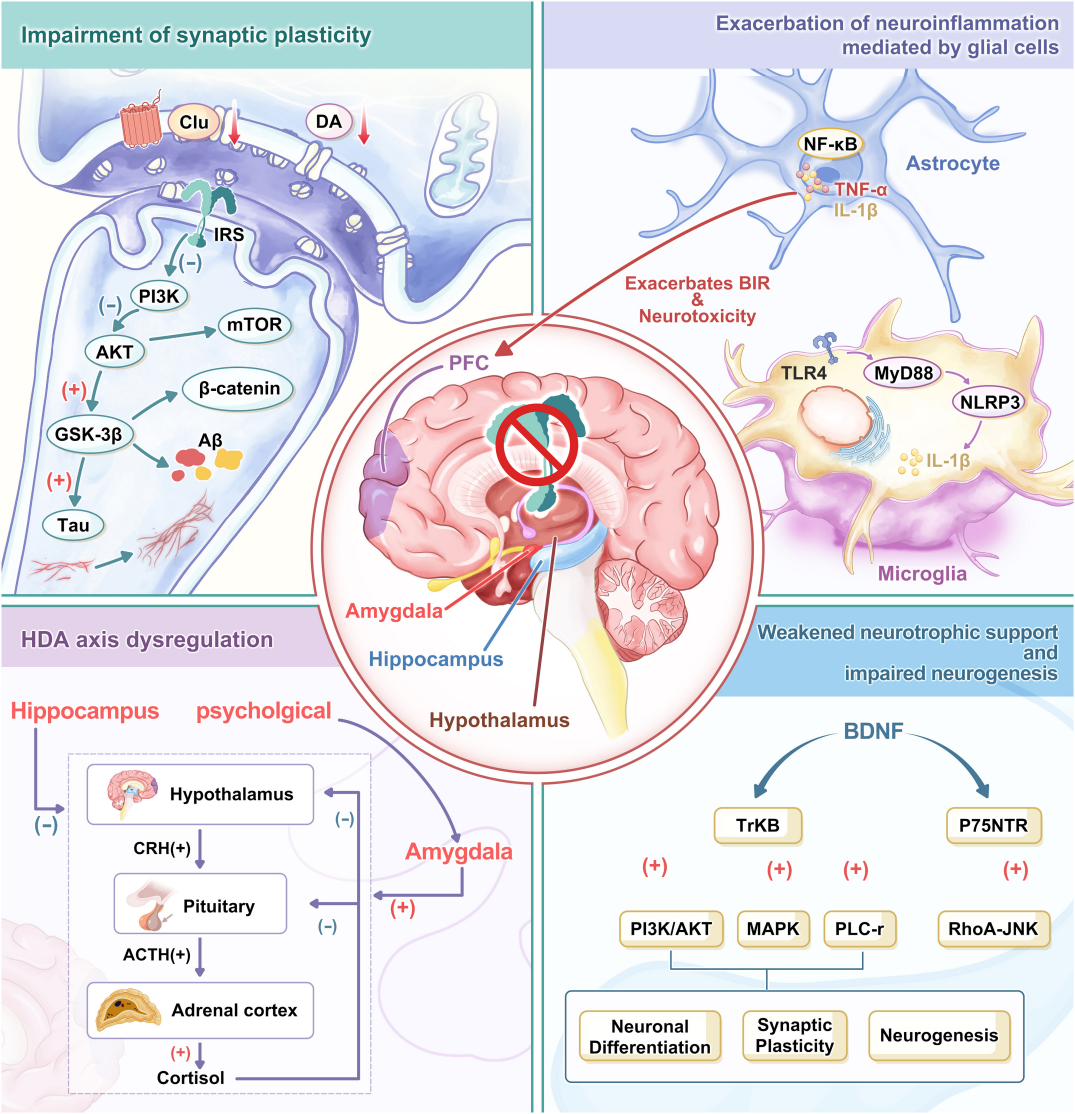
Mechanisms linking BIR to neuropsychiatric comorbidities. BIR can influence brain function by affecting the hypothalamus, amygdala, hippocampus, and prefrontal cortex (PFC). As a central pathological hub, BIR disrupts neural function through four interrelated mechanisms: impaired synaptic plasticity, heightened neuroinflammation, diminished neurotrophic support, and HPA axis dysregulation. In impaired synaptic plasticity, disrupted insulin signaling promotes abnormal tau phosphorylation and β-amyloid (Aβ) deposition by inhibiting the PI3K/Akt/mTOR pathway and overactivating GSK-3β kinase, ultimately leading to synaptic dysfunction and neuronal impairment. In neuroinflammation, disruption of the BBB allows saturated FAs to activate the TLR4-MyD88-NLRP3 pathway in microglia, promoting the release of proinflammatory cytokines like IL-1β and exacerbating BIR symptoms and neurotoxicity. Dysfunction in BDNF-TrkB signaling disrupts PI3K/Akt and MAPK/ERK pathways, thereby impairing neuronal differentiation, synaptic plasticity, and neurogenesis. The precursor form of BDNF, proBDNF, binds to the p75NTR receptor, activating the RhoA-JNK pathway to promote apoptosis and inhibit synaptic regeneration. Dysfunction of the HPA axis leads to sustained elevation of cortisol levels, exacerbating IR. Psychological stress activates the HPA axis via the amygdala-hypothalamic pathway, increasing cortisol levels and enhancing amygdala activity. These processes form a vicious cycle that ultimately results in cognitive deficits, depression, anxiety, and neurodegenerative pathology.

At the synaptic level, BIR impairs the induction and maintenance of LTP, the molecular basis for learning and memory. Studies indicate that BIR impairs NMDA receptor function, reducing calcium influx and inhibiting LTP formation (Shonesy et al., 2012; Spinelli et al., 2017). Certain agents, such as melatonin (Wang et al., 2005) and GLP-1 receptor agonists (Wang et al., 2021), alleviate impaired LTP, protect hippocampal and cortical neurons, and slow cognitive decline. Moreover, BIR alters dendritic spine morphology and density (Kellar and Craft, 2020; Jo et al., 2021), reducing synaptic connections and neural network stability. Thus, BIR impairs synaptic plasticity and neuronal function by suppressing PI3K/Akt/mTOR signaling and overactivating GSK-3β, ultimately leading to cognitive and neuropsychiatric disorders (Figure 1).

### 3.2 Exacerbation of neuroinflammation

Emerging evidence suggests that peripheral IR and chronic inflammation compromise BBB integrity, promote neurotoxicity and microglial activation, trigger neuroinflammation, and impair neuronal insulin signaling (Ponce-Lopez, 2025). Microglia, the primary immune cells in the CNS, become activated within days after exposure to a high-fat diet (HFD) (Lin et al., 2023). Saturated fatty acids (FAs) in the HFD interact mainly with Toll-like receptor 4 (TLR4) on microglia (Milanski et al., 2009; Hemmati et al., 2014), triggering assembly and activation of the NLRP3 inflammasome via the MyD88-dependent signaling pathway (Latz and Duewell, 2018). The activated NLRP3 inflammasome promotes caspase-1 activation, facilitating the maturation and release of pro-inflammatory cytokines such as IL-1β (Swaroop et al., 2018), thus amplifying neuroinflammation. This inflammatory cascade intensifies significantly during BIR, exacerbating the pathological progression (Komleva et al., 2021).

In hyperinsulinemia, astrocytes also exhibit marked inflammatory responses. Research indicates that hyperinsulinemia induces nuclear translocation of NF-κB in astrocytes, increasing synthesis and release of inflammatory mediators (Douglass et al., 2017; Lin et al., 2023). As a crucial transcription factor, NF-κB activation directly regulates inflammatory gene expression and further amplifies inflammation by interacting with other signaling pathways. Astrocyte inflammatory activation contributes to BIR-related neurodegenerative diseases such as AD (Akhtar et al., 2020b) and PD (Li et al., 2024). Additionally, activated astrocytes release neurotransmitters like glutamate, adversely affecting neuronal function and survival, creating a vicious cycle (Andersen et al., 2021).

The JNK pathway, a central mediator in inflammatory signaling, is considered an essential link between BIR and neuroinflammation. JNK, a member of the mitogen-activated protein kinase (MAPK) family, participates in numerous physiological processes, including cell proliferation, apoptosis, nervous system development, and T cell-mediated immunity (Davis, 2000). In IR, inhibition of insulin signaling pathways results in JNK overactivation, which phosphorylates downstream targets, enhancing the transcription and release of inflammatory cytokines (Devarajan et al., 2011). This process is particularly critical in AD, where JNK-mediated tau phosphorylation and mitochondrial dysfunction represent key neuronal degeneration factors (Khandelwal et al., 2020).

Insulin resistance also impairs mitochondrial function, resulting in decreased ATP production, ROS accumulation, inflammasome activation, NF-κB pathway activation, and increased inflammatory factor release (Kim et al., 2008). This mitochondrial inflammation intensifies neuroinflammation, impairs synaptic plasticity, and contributes to cognitive dysfunction and neurodegeneration (Qin et al., 2024).

Activated JNK signaling, triggered by BIR, not only regulates inflammatory cytokine release but also aggravates neuroinflammation and neuronal degeneration via mitochondrial dysfunction (Khandelwal et al., 2020).

### 3.3 Disruption of the BDNF pathway

BDNF is critical for hippocampal neurogenesis and synaptic plasticity (Yang et al., 2022). BDNF, widely expressed in the CNS, activates downstream signaling pathways such as PI3K/Akt, MAPK/ERK, and PLC-γ through its receptor TrkB. These pathways critically regulate neuronal survival, synaptic plasticity, and neurogenesis (Sasi et al., 2017). Dysfunctional signaling of BDNF and its receptor TrkB, along with disrupted PI3K/Akt and MAPK/ERK pathways, is a significant mechanism by which BIR impairs neuropsychiatric functions (Bumb et al., 2021). Animal studies indicate that hippocampal BDNF overexpression alleviates depressive behaviors in post-stroke rats (Chen et al., 2015). Patients with severe depression exhibit abnormally low serum BDNF levels, which increase following antidepressant treatment (Sen et al., 2008). Reduced BDNF levels in AD result primarily from Aβ peptide-induced inhibition of BDNF maturation, causing neurodegeneration and synaptic loss (Tanila, 2017; Amidfar et al., 2020). Additionally, reduced BDNF disrupts synaptic function by impairing the balance of glutamatergic neurons (Wang et al., 2024). Insulin-like growth factor-1 (IGF-1) enhances BDNF signaling, and synergizes with BDNF to produce antidepressant effects (Gururajan et al., 2016). Conversely, proBDNF, the precursor form of BDNF, binds to p75NTR receptors, activates the RhoA-JNK pathway, promotes apoptosis, and inhibits synaptic regeneration (Yang et al., 2021). The dual roles of BDNF signaling further underscore its complexity and importance in neuropsychiatric disorders.

Neurogenic impairment is another critical consequence of disrupted BIR and BDNF signaling pathways. Neurogenesis primarily occurs in the dentate gyrus region of the hippocampus (Spinelli et al., 2020), which is crucial for cognitive function and emotional regulation. Both insulin and BDNF significantly promote neurogenesis. Insulin activates PI3K/Akt and MAPK/ERK pathways, supporting neural stem cell proliferation and differentiation (Zhang et al., 2020). BDNF maintains neurogenic homeostasis by enhancing synaptic plasticity and neuronal survival (Bathina and Das, 2015). However, when BIR and BDNF signaling are impaired, neurogenesis becomes significantly reduced, causing hippocampal dysfunction and cognitive decline (Arnold et al., 2018).

It is reported that the disturbances in BIR and BDNF signaling pathways exacerbate neuropsychiatric disorders by affecting neuroinflammation and energy metabolism. Studies demonstrate that BIR reduces cerebral glucose uptake and glycogen synthesis, resulting in insufficient neuronal energy supply (Spinelli et al., 2020) and intensifying neurodegeneration through inflammatory responses. In AD pathology, BIR correlates closely with Aβ deposition and hyperphosphorylation of tau protein (Salkovic-Petrisic and Hoyer, 2007), and accelerating cognitive decline. Furthermore, BDNF mitigates neuroinflammation by inhibiting the HMGB1/RAGE/NF-κB pathway, protecting neurons from inflammatory damage (Han et al., 2019). However, in cases of disrupted BIR and BDNF signaling, this protective effect diminishes, explaining sustained neuroinflammatory activation and exacerbated neuronal injury.

Disruption of BDNF signaling pathways further intensifies these processes, accelerating neurodegenerative disease progression through impaired synaptic plasticity, neurogenesis, and neuroinflammatory responses (Song, 2024).

### 3.4 Dysregulated HPA axis

The HPA axis is a central component of the neuroendocrine system, responsible for regulating stress responses (Smith and Vale, 2006). Under physiological conditions, activation of insulin signaling in brain regions such as the hippocampus and PFC enhances glucocorticoid receptor (GR) function. This mechanism effectively inhibits excessive HPA axis activation and maintains cortisol homeostasis. However, BIR impairs this regulatory pathway, causing dysfunction of the HPA axis.

Abnormal activation of the HPA axis leads to persistent elevation of cortisol levels. Elevated cortisol further promotes hepatic gluconeogenesis, lipolysis, and inflammatory pathway activation (Björntorp et al., 1999). This exacerbates systemic IR and central obesity, creating a metabolic vicious cycle. It is evident that the HPA axis is activated in T2D patients, associated with cognitive impairment in diabetes. For example, a clinical study involving 1,066 elderly patients with T2D found a significant correlation between high morning cortisol levels and cognitive decline (Reynolds et al., 2010).

In addition, a close interaction exists between the HPA axis and emotional and rewards regulatory systems. Psychological stress activates the HPA axis via the amygdala-hypothalamus pathway. Increased cortisol levels provide positive feedback to enhance amygdala activity, further intensifying stress responses (Ulrich-Lai and Herman, 2009; Krämer et al., 2017). The dorsomedial PFC (DMPFC) and hippocampus normally exert negative feedback control over the HPA axis through inhibitory inputs (Herman et al., 2016), promoting emotional self-regulation during stress. Obesity increases T2D risk and disrupts the HPA axis by inhibiting hippocampal GRs (Pali et al., 2021; Lengton et al., 2025). Chronic elevation of glucocorticoids stimulates appetite by inhibiting CRH and promoting neuropeptide Y (NPY) expression (Kyrou et al., 2006). Furthermore, rewards pathways in the limbic system undergo neuroadaptive changes under prolonged exposure to high-fat, high-sugar diets, resulting in compulsive eating behaviors. Cessation of such diets activates the HPA axis, exacerbating emotional and metabolic disturbances (Lin et al., 2020).

In summary, BIR not only directly leads to HPA axis dysregulation but also initiates a vicious multisystem cycle involving metabolism, stress, and rewards mechanisms. This cycle further promotes cognitive dysfunction, obesity, and emotional disorders. Therapeutic strategies targeting HPA axis regulation, including cortisol suppression and neurofeedback interventions, may represent promising directions for treating these conditions.

## 4 Imbalanced neural circuits caused by BIR

The PFC facilitates judgment and decision-making through extensive neural connectivity. It supports higher-order cognitive functions, enabling advanced cognition, self-control, and judgment. Compared to healthy controls, functional connectivity analysis in AD patients revealed enhanced coupling between the left dorsolateral PFC, posterior cingulate cortex, and medial PFC. These connectivity changes correlated with cognitive performance (Zhao et al., 2022).

Neural circuits linking the PFC and limbic system are critical for emotion regulation, particularly in depression and anxiety disorders. Amygdala hyperactivation is a hallmark of depression and anxiety. Under normal conditions, glutamatergic projections from the PFC regulate amygdala excitability through the CRH system, suppressing excessive emotional responses (Gold, 2021b; Kim et al., 2022). However, in depressive disorders, persistent activation of central glutamate, norepinephrine, and cytokine systems, along with impaired PFC function, sustains anxiety and activates the amygdala, CRH/HPA axis, and sympathetic nervous system (Gold, 2015, 2021a). Reduced PFC activity and enhanced amygdala activity correlate with emotional disorder severity (Gold, 2021a).

Brain insulin resistance significantly impairs this regulatory mechanism, resulting in abnormal emotion regulation and triggering depression and anxiety symptoms. BIR reduces PFC neuronal sensitivity to insulin, impairing their function (Gold, 2015). Specifically, BIR weakens the PFC inhibitory control of the amygdala by suppressing synaptic plasticity and neurotransmitter release. Clinical studies have found weakened prefrontal inhibition of amygdala activation in obese, depressed young patients, closely linked to BIR (Sun et al., 2018). Additionally, BIR-induced disruptions in other neurotransmitter systems and neural circuits exacerbate emotional dysregulation (Kleinridders et al., 2014).

In summary, BIR reduces inhibitory control by the PFC over the amygdala, leading to abnormal emotional regulation, which is an important pathological mechanism underlying depression and anxiety.

## 5 BIR in metabolic and neuropsychiatric disorders

Brain insulin resistance commonly arises from early hypothalamic inflammation induced by a HFD, preceding inflammation in peripheral tissues (Jais and Brüning, 2017). Chronic overnutrition triggers persistent hypothalamic inflammation through interactions between neuronal and non-neuronal cells, making initially reversible processes permanent. This ultimately results in disrupted calorie intake and energy expenditure regulation, promoting overeating and weight gain.

In animal model studies, HFD induces significant peripheral IR and BIR in T2D mouse models (Mengr et al., 2023), accompanied by behavioral phenotypes resembling depression, anxiety, and cognitive impairment (Reagan et al., 2021). These animals demonstrate significantly increased immobility in forced swimming and tail suspension tests, reduced sucrose preference, and decreased open-arm duration in elevated plus maze tests, indicating heightened anxiety-like behavior.

Clinical epidemiological evidence indicates that the prevalence of depression is higher among T2D patients, and comorbidity between depression and diabetes is frequent (Moussavi et al., 2007). Diabetic complications further exacerbate depression, particularly in poorly controlled diabetes (Mezuk et al., 2008). Elderly diabetic patients also frequently experience MCI. Individuals with T2D, impaired fasting glucose, or metabolic syndrome exhibit diminished memory, processing speed, and executive function compared to healthy controls (Koekkoek et al., 2015).

Diabetes-associated cognitive decline (DACD) represents one of the most direct neuropsychiatric disorders correlating with BIR, characterized by marked reductions in information processing speed, executive function, and psychomotor efficiency. These clinical features correspond closely to dysfunction in brain regions highly sensitive to insulin, particularly the PFC and hippocampus (Moheet et al., 2015). Pathologically, BIR induces cerebrovascular endothelial dysfunction, reduces cerebral blood flow, and leads to white matter hyperintensities (Bangen et al., 2018).

Regarding emotional disorders, excessive activation of IR, HPA axis dysregulation, and neuroinflammation create a vicious cycle that contributes to chronic loss of pleasure and other depressive symptoms, correlating with a twofold increased risk of depression among patients with T2D compared to the general population. Even non-diabetic patients experiencing depression exhibit elevated BIR markers, indicating that BIR is a depression risk factor (Nasca et al., 2021; Watson et al., 2021). Comorbid depression further accelerates cognitive decline in diabetic patients, creating a challenging cycle of comorbidity.

In AD patients, cerebrospinal fluid (CSF) insulin levels and insulin-mediated glucose metabolism are decreased (Stanley et al., 2016). Autopsy studies on AD brains reveal coexisting BIR and β-amyloid deposition, implicating BIR in AD-related cognitive deficits (Frölich et al., 1998). General anesthesia is believed to be a risk factor for AD, and Morris water maze and novel object recognition tests reveal impaired spatial and recognition memory, suggesting severe cognitive dysfunction (Zhang et al., 2016). Enhancing insulin sensitivity in these brain regions improves behavioral phenotypes, while intranasal insulin is considered a promising treatment for AD and MCI (Shpakov et al., 2023).

As Alzheimer’s-like pathology progresses, DACD may evolve into typical AD, also termed “type 3 diabetes” (Kandimalla et al., 2017). Numerous autopsy studies indicate abnormal IRS-1 phosphorylation in AD brains, a hallmark of BIR strongly correlated with tau pathology severity, further supporting BIR and its direct link to AD pathology (Frölich et al., 1998). Epidemiological studies also strongly support T2D as an important risk factor for AD, increasing the risk by approximately 1.5–2.0 times (Strachan et al., 2011), strongly suggesting that interventions targeting BIR may alleviate AD pathology and severity.

The association between BIR and PD is also attracting growing attention. Epidemiological studies indicate a reciprocal risk relationship between T2D and PD. Mechanistically, mitochondrial dysfunction, oxidative stress, and chronic neuroinflammation associated with BIR exacerbate metabolic stress in dopaminergic neurons of the substantia nigra pars compacta. These factors may promote abnormal α-synuclein aggregation, accelerating PD pathology (AlRuwaili et al., 2025).

In summary, BIR induces neuropsychiatric changes primarily by damaging brain regions such as the hypothalamus, hippocampus, amygdala, and PFC. These effects significantly influence pathological mechanisms in various disorders. BIR serves as a critical common pathological foundation linking metabolic diseases and neuropsychiatric comorbidities. An in-depth exploration of BIR’s pathological mechanisms may further elucidate intrinsic connections between these disorders and provide a robust theoretical basis for developing novel therapeutic strategies (Table 1).

**TABLE 1:** Summary of BIR-related findings in neuropsychiatric disorders and metabolic cognitive impairment.

Disorder	Model type	Key outcomes related to BIR	References
AD	Human (postmortem, CSF); Animal (ICV-STZ, HFD)	↓ CSF insulin; ↑ Aβ and p-tau; impaired hippocampal synaptic plasticity; IRS-1 serine phosphorylation	Stanley et al., 2016; Frölich et al., 1998; Tramutola et al., 2015; Shonesy et al., 2012; Spinelli et al., 2017
DACD	Human (T2D patients); Animal (HFD, T2D models)	↓ Processing speed, executive function; ↑ white matter hyperintensities; cerebrovascular endothelial dysfunction	Moheet et al., 2015; Bangen et al., 2018
PD	Animal and cellular models	Mitochondrial dysfunction; oxidative stress; α-synuclein aggregation; PI3K/Akt/GSK-3β pathway disruption	Kim et al., 2008; AlRuwaili et al., 2025
Depression	Human (clinical, biomarker); Animal (HFD, NIRKO)	↓ PFC inhibition of amygdala; ↓ hippocampal BDNF; peripheral IR markers	Sun et al., 2018; Chen et al., 2015; Nasca et al., 2021; Watson et al., 2021
Anxiety	Animal (HFD, hippocampal-specific IR)	↑ Amygdala hyperactivity; ↓ PFC regulatory function; despair- and anxiety-like behaviors; dendritic atrophy	Gold, 2015, 2021a; Sun et al., 2018; Reagan et al., 2021; Jo et al., 2021

BIR, brain insulin resistance; CSF, cerebrospinal fluid; ICV-STZ, intracerebroventricular streptozotocin; HFD, high-fat diet; Aβ, amyloid-beta; p-tau, phosphorylated tau; IRS-1, insulin receptor substrate 1; HPA, hypothalamic-pituitary-adrenal; PFC, prefrontal cortex; BDNF, brain-derived neurotrophic factor; IR, insulin resistance; NIRKO, neuron-specific insulin receptor knockout. ↑ indicates an increase in content; ↓ indicates a decrease in content.

## 6 Intervention strategies

The increasing recognition of BIR as a common pathological mechanism linking metabolic and neuropsychiatric comorbidities has prompted a shift from traditional symptomatic management to therapies targeting underlying metabolic dysfunction. Current intervention strategies broadly include lifestyle modifications (McAuley et al., 2002), repurposed anti-diabetic medications (Cha et al., 2016), and novel targeted treatments. These interventions primarily aim to enhance brain insulin sensitivity and improve associated disorders.

Lifestyle modification represents a fundamental intervention. Studies indicate that adherence to a Mediterranean diet or caloric restriction reduces microglial activation (Hornedo-Ortega et al., 2018; Folch et al., 2019), thereby mitigating neuroinflammation and positively impacting brain function. Exercise, particularly aerobic training, also offers clear benefits. It enhances peripheral glucose metabolism (Hopkins et al., 2020) and directly improves brain insulin signaling (Kullmann et al., 2022a; Cai et al., 2024).

Pharmacological approaches increasingly emphasize the CNS effects of anti-diabetic drugs. Metformin, a first-line insulin sensitizer, has been shown in animal models to enhance hippocampal insulin signaling (pAkt/Akt ratio) under conditions of diet-induced IR (Barzilai et al., 2016). Metformin has also been shown to reduce microglial activation, restore autophagic flux, increase synaptic protein expression, and improve anxiety-like behavior (Ai et al., 2020; González Pérez et al., 2025). *In vitro* studies further confirm that metformin reverses palmitate-induced neuroinflammation and mitochondrial dysfunction (Ruegsegger et al., 2019; Lu et al., 2020; González Pérez et al., 2025). These central benefits complement its well-documented peripheral insulin-sensitizing and neuroprotective effects.

Glucagon-like peptide-1 (GLP-1) receptor agonists, such as liraglutide, currently have strong supporting evidence. These drugs cross the BBB to activate GLP-1 receptors, thereby enhancing insulin signaling (Li et al., 2008), reducing neuroinflammation (Qian et al., 2022), and promoting synaptic plasticity and neuronal survival (Salcedo et al., 2012). A small, non-placebo-controlled clinical trial involving GLP-1 receptor agonist liraglutide treatment in 19 non-diabetic patients with emotional disorders reported improved cognitive function (Mansur et al., 2017). Additionally, other GLP-1 receptor agonists, including semaglutide, are currently undergoing phase III clinical trials (EVOKE/EVOKE+) to evaluate their efficacy in the early stages of AD. These trials use declines in cognitive function and daily activities as primary endpoints, establishing associations between the biological effects of the drugs and clinical outcomes through exploratory plasma biomarkers (Cummings et al., 2025).

In addition to GLP-1 receptor agonists, intranasal insulin is another method that bypasses the BBB to deliver insulin directly to the brain (Fan et al., 2019). By elevating intracerebral insulin levels, this approach directly enhances central insulin signaling, thereby counteracting key pathological processes associated with BIR. It holds dual potential for metabolic and cognitive health (Raza et al., 2025). Clinical trials in patients with MCI and AD have demonstrated improvements in verbal memory and overall cognitive function (Claxton et al., 2015; Avgerinos et al., 2018), though high-dose administration may induce BIR (Zakharova et al., 2021).

SGLT2 inhibitors inhibit sodium-glucose transport proteins in the kidney and therefore lower blood sugar. SGLT2 inhibitors, such as empagliflozin, have shown evidence of directly improving hypothalamic insulin sensitivity in humans. A randomized controlled trial in individuals with prediabetes found that 8 weeks of treatment with 25 mg/day of empagliflozin significantly restored hypothalamic insulin sensitivity, as measured by the fMRI response to intranasal insulin. This improvement mediated reductions in fasting glucose, reductions in intrahepatic lipid content, and decreases in adipose tissue mass (Kullmann et al., 2022b). While SGLT2 inhibitors are not classic insulin sensitizers, their ability to improve hypothalamic insulin sensitivity underscores a key principle: systemic metabolic interventions can exert meaningful effects in the brain. This provides a strong rationale for further exploring established insulin-sensitizing agents, especially those with central penetration, as potential treatments for neuropsychiatric disorders.

While direct evidence measuring brain insulin sensitivity following interventions remains technically challenging and therefore limited, a compelling body of indirect evidence suggests that strategies improving peripheral IR also ameliorate BIR-related neuropathologies and clinical outcomes. These findings support the premise that targeting systemic metabolism is a viable strategy for addressing brain disorders (Table 2).

**TABLE 2:** Evidence summary of selected interventions targeting BIR and their effects on related phenotypes.

Intervention	Direct evidence for BIR improvement	Evidence for reversing BIR-related phenotypes	Evidence as peripheral insulin sensitizer	References
Metformin	Strong (animal model) ↑ Hippocampal pAkt/Akt ratio in HFD mice	Strong ↓ Microglial activation ↑ Synaptophysin ↓ Anxiety-like behavior *In vitro*: ↓ IL-1β, TNFα	Well-established First-line for T2D ↑ Hepatic insulin signaling	Barzilai et al., 2016; Ruegsegger et al., 2019; Ai et al., 2020; Lu et al., 2020; González Pérez et al., 2025
GLP-1 RAs (semaglutide)	Indirect (ongoing RCT) EVOKE trials in early AD CSF biomarkers of neuroinflammation and synaptic integrity	Strong (planned outcomes) Primary: slow cognitive/functional decline Exploratory: ↓ Plasma NfL, p-tau181, GFAP	Very strong Well-established for T2D/obesity ↓ HbA1c, body weight, inflammation	Cummings et al., 2025
SGLT2 inhibitors (empagliflozin)	Strong (human RCT) ↑ Hypothalamic insulin sensitivity (fMRI) 8-weeks treatment in prediabetes	Moderate ↓ Fasting glucose ↓ Intrahepatic liver fat ↓ Adipose tissue mass	Mechanistically distinct Promotes urinary glucose excretion No change in HOMA-IR	Kullmann et al., 2022b

## 7 Conclusions and future directions

In this review, we have outlined the growing evidence for the critical role of BIR in neuropsychiatric disorders. A comprehensive analysis of clinical and preclinical evidence has identified BIR as a central pathological link connecting metabolic dysfunction to neuropsychiatric comorbidities, including depression, anxiety, and cognitive impairment. BIR impacts neural function through multiple pathological mechanisms, including: (1) significant impairment of synaptic plasticity; (2) intensified neuroinflammation; and (3) disrupted mitochondrial function within critical limbic circuits. These pathological changes collectively form a neurobiological foundation that crosses traditional diagnostic boundaries. Notably, a bidirectional relationship exists between BIR and neurological dysfunction, forming a positive feedback loop that may explain the high comorbidity rates observed between metabolic diseases and neuropsychiatric disorders (Table 3).

**TABLE 3:** Summary of key findings related to BIR and available treatment options for improving BIR.

Disease	Primary pathway	Outcome/phenotype	Targeted BIR intervention
AD	Impaired PI3K/Akt/mTOR signaling; Excessive activation of GSK-3β; Abnormal serine phosphorylation of IRS-1	↓ CSF insulin; ↑ Aβ and p-tau; Impaired hippocampal synaptic plasticity/cognitive decline	Lifestyle intervention; GLP-1 RA; Intranasal insulin
MCI	Impaired insulin signaling (PI3K/Akt) related to hippocampal synaptic plasticity	Impaired verbal memory and overall cognitive function	Lifestyle intervention; GLP-1RA; Intranasal insulin
DACD	Impaired function in insulin-sensitive brain regions (PFC/hippocampus); Cerebral vascular endothelial dysfunction, white matter hyperintensity	Decreased information processing speed/executive function; High white matter signal intensity; cognitive decline	Lifestyle intervention; Metformin; SGLT2 inhibitors
PD	Mitochondrial dysfunction/oxidative stress/chronic inflammation; Disruption of the PI3K/Akt/GSK-3β pathway	α-Synuclein aggregation, exacerbated neuronal metabolic stress	Lifestyle interventions; GLP-1 RA
Depressive disorders	Reduced PFC inhibition of the amygdala; Decreased hippocampal BDNF; Hyperactive HPA axis/amplified inflammation	Abnormal emotion regulation and depressive symptoms; Increased depression risk in T2D populations, potentially accelerating cognitive decline	Lifestyle interventions; Metformin; GLP-1 RA
Anxiety disorders	Hyperactive amygdala, impaired PFC regulation; Dendritic alterations/impaired plasticity associated with BIR	Anxiety/despair-like behaviors, dendritic atrophy	Lifestyle interventions; Metformin
Obesity/HFD-related BIR	Early hypothalamic inflammation; TLR4/MyD88/NLRP3 inflammatory response	Imbalance in energy intake/expenditure regulation, overeating, and weight gain; Accompanied by depression/anxiety-like symptoms and cognitive impairment phenotypes	Lifestyle interventions; Metformin; GLP-1RA; SGLT2 inhibitors

CSF, cerebrospinal fluid; Aβ, amyloid-beta; p-tau, phosphorylated tau; PFC, prefrontal cortex; BDNF, brain-derived neurotrophic factor.

It is plausible to hypothesize that any other diagnosis such as depression, anxiety or MCI that is associated with a major metabolic disturbance (IR, obesity, etc.) has one more subtype (the BIR-positive type). The essential features of this subtype may include hyperactivation of the HPA axis, increased levels of inflammatory markers, and poor response to standard antidepressants but a better response to insulin sensitizers and anti-inflammatory agents. Future investigations to establish the presence of the above subtype at the clinical and molecular levels have the potential to alter our psychiatric classification system and enhance precision psychiatry.

Brain insulin resistance plays an important role in neuropsychiatric diseases, serving as one of the key drivers of cognitive and psychiatric disorders. It would be beneficial to develop cost-effective and reliable BIR biomarkers for early identification and intervention. So far, various biomarkers have been suggested to assess IR, like fasting-insulin levels, A-FABP, and the TyG index. However, none of those known biomarkers are specific indicators of BIR. Practical and non-invasive central biomarkers for BIR are worthy of investigation.

Although there are multiple candidate intervention strategies, we lack sufficient clinical experience and knowledge about BIR-targeting strategies in patients with the allegedly “metabolic neuropsychiatric syndrome” subtype. Large-scale randomized controlled trials exclusively focused on BIR-targeting strategies are required in future clinical studies.

Furthermore, a more thorough and accurate understanding of BIR’s pathological mechanisms is poised to provide precise treatment strategies for patients with treatment-resistant neuropsychiatric disorders and high metabolic risk, ultimately paving the way for a new era of metabolic psychiatry.
